# A methodological approach of QRA for slow-moving landslides at a regional scale

**DOI:** 10.1007/s10346-022-01875-x

**Published:** 2022-04-02

**Authors:** Francesco Caleca, Veronica Tofani, Samuele Segoni, Federico Raspini, Ascanio Rosi, Marco Natali, Filippo Catani, Nicola Casagli

**Affiliations:** 1 Department of Earth Sciences, University of Florence, via La Pira 4, Florence, Italy; 2 Department of Geosciences, University of Padova, Via Gradenigo 6, Padova, Italy

**Keywords:** Slow-moving landslides, QRA, Landslide risk, Vulnerability, Arno River basin

## Abstract

Landslides represent a serious worldwide hazard, especially in Italy, where exposure to hydrogeological risk is very high; for this reason, a landslide quantitative risk assessment (QRA) is crucial for risk management and for planning mitigation measures. In this study, we present and describe a novel methodological approach of QRA for slow-moving landslides, aiming at national replicability. This procedure has been applied at the basin scale in the Arno River basin (9100 km^2^, Central Italy), where most landslides are slow-moving. QRA is based on the application of the equation risk = hazard (H) × vulnerability (V) × exposure (E) and on the use of open data with uniform characteristics at the national scale. The study area was divided into a grid with a 1 km^2^ cell size, and for each cell, the parameters necessary for the risk assessment were calculated. The obtained results show that the total risk of the study area amounts to approximately 7 billion €. The proposed methodology presents several novelties in the risk assessment for the regional/national scale of the analysis, mainly concerning the identification of the datasets and the development of new methodologies that could be applicable over such large areas. The present work demonstrates the feasibility of the methodology and discusses the obtained results.

## Introduction

Italy is a country where the exposure to the risk of hydrogeological disasters is very high; landslides and mass movement are very common in Italy (Catani et al. 2005). They are the natural hazards that occur with the highest frequency, and after earthquakes, they cause the most casualties and damage to buildings and infrastructure.

Risk analysis is a process of estimating the risk of landslide hazards to populations and property. This process can facilitate financial and cost–benefit analyses when planning risk reduction strategies (Fell et al. 2008). There are three types of risk analysis: qualitative, semiquantitative, and quantitative (Chowdhury and Flentje 2003).

Quantitative risk assessment (QRA) is distinguished from qualitative risk analysis by the input data, the procedures used in the analysis and the final risk output. In contrast with qualitative risk analysis, which yields results in terms of weighted indices, relative ranks (e.g. low, moderate, and high) or numerical classification, QRA quantifies the probability of a given level of loss and the associated uncertainties (Corominas et al. 2013). QRA is important for scientists and engineers because it allows risk to be quantified in an objective and reproducible manner, and the results can be compared from one location (site, region, etc.) to another. For landslide risk managers, it is also useful because it allows a cost–benefit analysis to be performed, and it provides a basis for the prioritization of management and mitigation actions and the associated allocation of resources. For society in general, QRA helps to increase the awareness of existing risk levels and the appreciation of the efficacy of the actions undertaken. QRA uses numerical values and mathematical methods to estimate objective probabilities. A quantitative risk map is produced on a continuous scale in which numerical values indicate the distribution of the risk expressed by the probability of the expected losses for the elements at risk. QRA is performed separately for each type of element (specific risk), and the results are then integrated into a map of the total risk by combining all maps of the specific risk (total risk).

According to the Varnes and IAEG Commission on Landslides (1984), risk is defined as *R* (*I*) = *H* × *V* (*I*) × *E*, where R is the landslide risk, H is the landslide hazard, V is the vulnerability of vulnerable elements, I is the intensity of landslides and E is the value of the element at risk (e.g. the number of people or the monetary value of the buildings).

Landslide hazard assessment means the estimation of the zones where landslides of a particular type, volume, runout and intensity may occur within a given period of time (Corominas et al. 2013). The evaluation of hazards can be based on the definition of the following probabilities: (*i*) spatial probability: the probability that a given area will be hit by a landslide, (*ii*) temporal probability: the probability that a given triggering event will cause landslides, (*iii*) size/volume probability: the probability that the slide has a given size/volume, (*iv*) reach probability: the probability that the slide will travel a certain distance downslope (Corominas et al. 2013). In particular, the spatial probability is also defined as landslide susceptibility.

Landslide susceptibility assessment can be considered the initial step towards landslide hazard and risk assessment, but it can also be a final product in itself that can be used in land-use planning and environmental impact assessment (Corominas et al. 2013). In landslide susceptibility maps (LSMs), each terrain unit is associated with a numerical index that represents the spatial probability of a landslide occurrence, but LSMs do not explicitly convey information about landslide return periods (Brabb 1984).

Intensity is used as a general term, which can include different concepts and parameters (Lari et al. 2014). Different researchers have tried to define the damaging capability of landslides in different ways and by using a variety of parameters. Intensity is essentially considered to depend upon kinetic energy (mass and velocity) (Hungr 1995) and geometric characteristics (Einstein 1988). Hungr (1997) argued that the maximum movement velocity is the most important intensity parameter and defined a scale of destructiveness based on the velocity and also provided specific velocity thresholds associated with the different intensity classes. Evans (2003) used the landslide area to set curves of the destructiveness of the landslide. Landslide volume has also been used by several researchers (Cardinali et al. 2002) as a proxy for landslide magnitude. In addition to velocity, extent and volume, other proposed parameters are represented by the recharge rate (Jakob et al. 2005), the depth of the deposit (Revellino et al. 2008) and the impact velocity (Luo et al. 2019) for debris flows and avalanches or trajectories and kinetic energy (Copons et al. 2005; Corominas et al. 2005; Jaboyedoff et al. 2005) for rock falls.

Despite the many suggested methods, the practical assessment of intensity is still a quite difficult task, since it is highly site-dependent (Corominas et al. 2014), and many parameters, both geometric and kinematic, should be accounted for, depending on the landslide type and propagation mechanism. A definition of the landslide intensity at the basin scale is even more challenging given the lack of information on the expected velocity for a large number of landslides and the assumptions necessary to estimate the volume for a large dataset of landslides (Catani et al. 2005; Lu et al. 2014).

Vulnerability is defined as the degree of loss of a given element or set of elements exposed to the occurrence of a landslide of a given magnitude/intensity. It is expressed on a scale of 0 (no loss) to 1 (total loss) (Varnes and IAEG Commission on Landslides 1984; Fell 1994; Corominas et al. 2014). The assessment of vulnerability involves in many cases the evaluation of several different parameters and factors, such as building materials and techniques, the state of conservation, the presence of protection structures and the presence of warning systems (Fell 1994; Fell and Hartford 1997). Vulnerability can be split into physical vulnerability and vulnerability of people. Physical vulnerability refers to the direct damage to buildings, utilities and infrastructure, while the vulnerability of people (fatalities, injuries) relates to whether a landslide event will result in injuries or fatalities.

The vulnerability of people depends on many factors, such as the landslide type, size and intensity; the resistance and mobility of the individuals affected by the landslide hazard; and their relative positions in the exposed area. The resistance of a person to landslides is believed to also be a function of the person’s intellectual maturity (e.g. perception of the risk) and physical ability (e.g. age) (Uzielli et al. 2008). Considering the large uncertainties and complexities associated with the vulnerability of people to landslides, all existing methodologies are based on expert judgement and empirical data.

Conversely, many studies have been proposed for the assessment of physical vulnerability in recent years (Crozier and Glade 2005; Peng et al. 2014, 2015; Uzielli et al. 2015; Ferlisi et al. 2021). The methodologies used for the quantification of vulnerability can be classified according to the type of input data and the evaluation of the response parameters into judgemental/heuristic, data-driven (using data from past events) or analytical (using physical models) (Corominas et al. 2014). However, the majority of the proposed approaches are related to site-specific and local scale analyses given the difficulties in evaluating sound and reproducible maps of vulnerability at the regional scale. Exposure, defined as the number of lives or the value of the exposed properties at risk, is often strictly connected to vulnerability in its practical assessment (Schuster and Fleming 1986; Schuster and Turner 1996).

Despite the impressive amount of work dealing with QRA and the latest advances in the field, it should be stressed that, to date, landslide QRA analyses are mainly limited to test sites with small area extensions. When working in very broad areas (e.g. entire nations or regions), QRA is hampered by the difficulty of gathering complete and homogeneous datasets and the impossibility of applying refined methodologies. In such cases, landslide risk studies usually rely on the definition of simplified indicators (Guillard-Gonçalves et al. 2015; de Almeida et al. 2016; Pereira et al. 2020; Iadanza et al. 2021; Segoni and Caleca 2021), which have the advantage of being very easy to apply, update and disseminate, but from a scientific point of view, they represent an oversimplification of a full QRA procedure.

The objective of this paper was to conceive, define and test a methodological approach for landslide QRA in terms of the expected damage to buildings and the land use applicable at the national scale for slow-moving landslides. The methodology has been applied and tested in the Arno River basin. The final purpose is to have a tool for land planning and prioritization of the economic resources for landslide disaster risk reduction.

## Materials and methods

### Methodological approach and input data

In this work, the risk for slow-moving landslides is defined as *R* (*I*) = *H* × *V* (*I*) × *E*, where R is the landslide risk, H is the landslide hazard, V is the vulnerability of vulnerable elements, I is the intensity of landslides and E is the value of the element at risk (e.g. the number of people or the monetary value of the buildings).

**Fig. 1:**
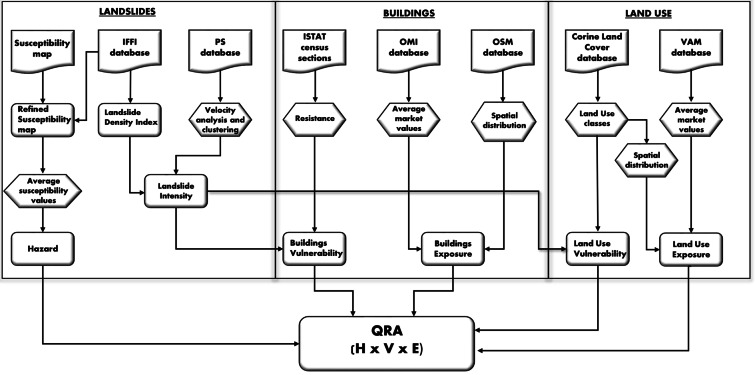
Flowchart of the proposed methodology for QRA

In particular, due to the difficulty of retrieving suitable information at the national scale for the evaluation of the temporal probability occurrence of landslides, which is usually expressed as frequency, return period, or exceedance probability, we decided to use the susceptibility (spatial probability of occurrence) instead of the hazard (spatial and temporal probability of occurrence). We are aware that this decision represents a simplification of the problem. However, as stated by Corominas et al. (2014) landslide susceptibility assessment can be considered an end product in itself that can be used in land-use planning and environmental impact assessment. This stands especially in small-scale analyses or in situations where sufficient information on past landslide occurrence is lacking, hampering the assessment of the spatial and temporal probabilities of events. In this work, we focused on the evaluation of the specific risk of the buildings and the land use classes. Since our objective was to create a procedure to be applied at the national scale, all of the input data used are open access, and they have uniform characteristics at the national scale (Table 1). The detailed procedure for the evaluation of QRA and of the single component of risk is described in Fig. 1. The resolution of the QRA map is 1 km^2^, and floodplains have been excluded from the analysis to simplify the calculation.

**Table 1:** The input data with their features and role in the QRA. The translation of the Italian acronyms is provided hereafter: *IFFI* (*Inventario Fenomeni Franosi in Italia*) stands for “inventory of Italian landslides”; *ISTAT* (*Istituto nazionale di STATistica*) stands for “National Institute of Statistics”; *OMI* (*Osservatorio Mercato Immobiliare*) stands for “real estate market observatory”; *VAM* (*Valori Agricoli Medi*) stands for “mean agricultural values”

Input data	Description	Risk parameter	Scale/resolution	Website/reference
DTM	Digital Terrain Model	Risk analysis mask	10 m	http://tinitaly.pi.ingv.it/
IFFI database	Database of Italian landslides	Hazard — intensity	1:10.000/1: 25.000	https://www.isprambiente.gov.it/it
Susceptibility map	Spatial probability of landslides occurrence	Hazard	50 m	Trigila et al. (2013)
PS database	Distribution of ground deformation and movement rate	Intensity	National	Raspini et al. (2018)
ISTAT census sections	Spatial distribution of buildings characteristics	Vulnerability	From 1:5.000 to 1:25.000	https://www.istat.it/
OMI database	Market value of buildings	Exposure	Sub-municipal scale	https://www.agenziaentrate.gov.it/
Open Street Map (OSM) database	Spatial distribution of buildings	Exposure	1:5.000	https://www.openstreetmap.org/
Corine Land Cover (CLC) database	Spatial distribution of land use	Vulnerability — exposure	European scale — minimum mapping unit (MMU) of 25 ha	https://www.isprambiente.gov.it/it
VAM database	Market value of land use	Exposure	Municipal scale	https://www.agenziaentrate.gov.it

The proposed methodology is presented and discussed through a case study of the Arno River basin (Central Italy), where every single step of the methodological approach was tested and calibrated.

### Study area

**Fig. 2:**
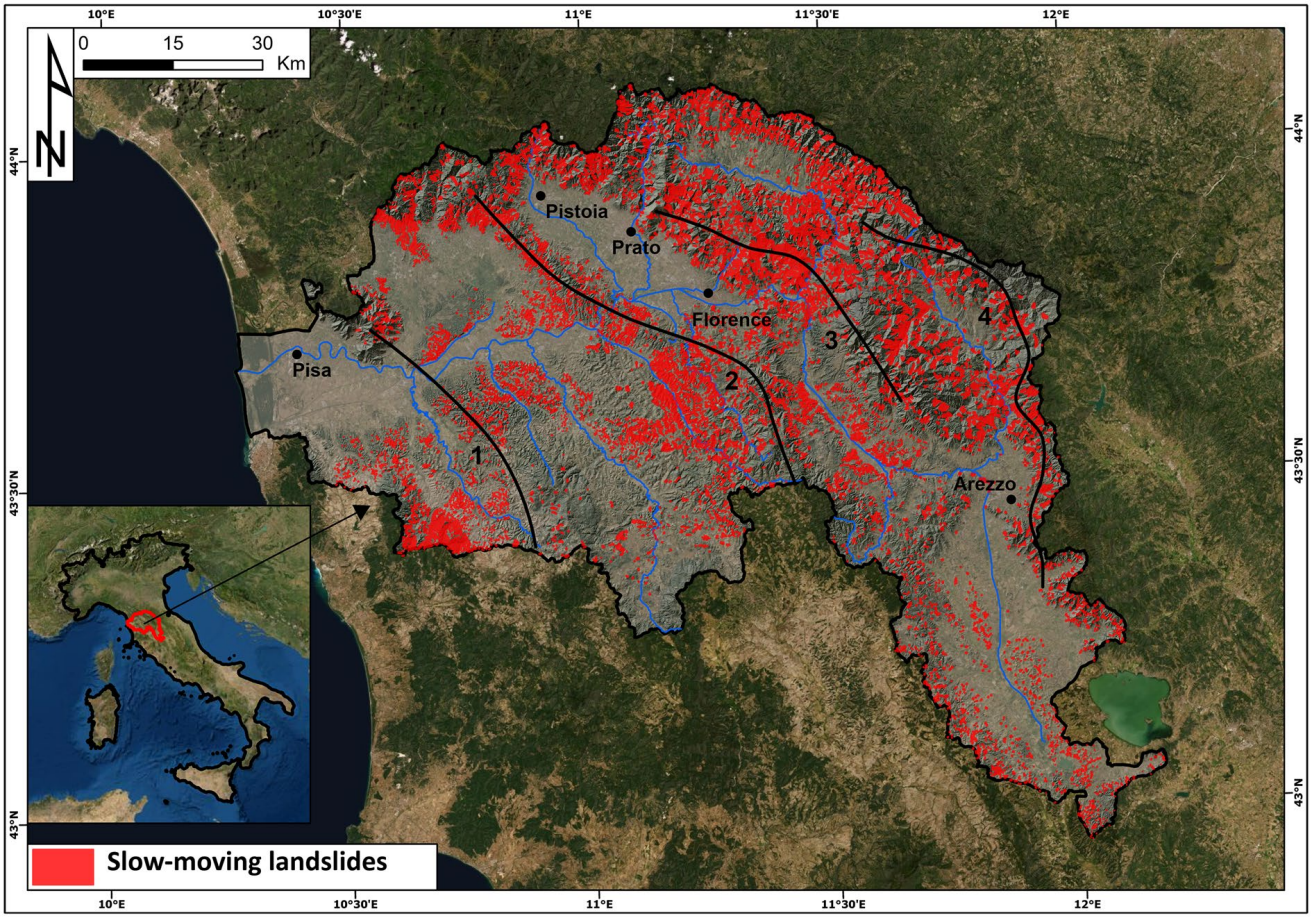
Location, main drainage, slow-moving landslides and major mountain ridges of the Arno River basin. (1) Mt-Pisano-Montagnola Senese; (2) Mt. Albano-Chianti; (3) Calvana-Mt. Morello-Pratomagno; (4) Falterona-Mandrioli-Alpe di Catenaia

The Arno River basin (Central Italy) extends for approximately 9100 km^2^ and is primarily located in the Tuscany region (98.4%) and for a small part in the Umbria region (1.6%) (Fig. 2). The Arno River basin is located on the inner side of the Northern Apennine chain, which is a complex thrust-belt system made up of the juxtaposition of several tectonic units built up during the Tertiary under a compressive regime that was followed by extensional tectonics from the Upper Tortonian. The extensional phase produced a sequence of horst-graben structures with an alignment NW–SE that have since been filled with marine (to the west) and fluvio-lacustrine (to the east) sediments (Vai and Martini 2001) set down from the Upper Tortonian to the Quaternary.

From a geomorphological point of view, the Arno River basin is mainly hilly, with four chains: Monti Pisani-Montagnola Senese, Monte Albano-Chianti, Calvana-Monte Morello and Pratomagno, Monte Falterona-Mandrioli-Alpe di Catenaia, mainly made up of flysch rocks. In the plains, cohesive and granular fluvio-lacustrine sediments crop out.

The area is characterized by a temperate climate with a dry summer. The general annual rainfall pattern is typified by a summer minimum in July and two maxima, one in November and the other at the end of the winter. Mean values of yearly rainfall vary in relation to relief and location, ranging from 800 mm on the Chiana valley to approximately 1800 mm on the Apennine ridges.

It is widely known and agreed that slides affecting the Arno River basin and generally the Northern Apennines mainly move by reactivation of dormant slides probably initiated during the early phases of the Holocene as a consequence of the sharp climate variation that began at the end of the last cold oscillation (Bertolini et al. 2004). The study area is strongly subjected to mass movements that have accumulated a large number of recorded cases and substantial total damage, both in properties and life losses. Landslides are very common in the study area. The geological settings and lithological characteristics of the area affect the typology and occurrence of landslides, which are mainly constituted by slow-moving rotational slides (IAEG Commission on Landslides 1990; Bertolini et al. 2004; Catani et al. 2005, 2013, 2016; Bicocchi et al. 2019).

According to the IFFI database, the Italian national inventory of landslides at a 1:10,000 scale (Trigila et al. 2010), 18,134 landslides were mapped in the Arno River basin. The majority are slides (87.4%), rotational and translational, followed by flows (6.3%), complex movements (2.1%) and rock falls/topples (1.7%). Rapid flows make up less than 1%, while more than 2% of the movement’s typology has not been defined. The areas of the phenomena range from 100 m^2^ to 5 × 10^6^ m^2^, and the average area is 3 × 10^4^ m^2^. In the Arno River basin, the majority of landslides are slow-moving (rotational and translational slides and complex movements) (Catani et al. 2005). According to Catani et al. (2005) rotational and translational slides affecting the Arno River basin and generally the Northern Apennines, mainly move by reactivation of dormant slides probably initiated during the early phases of the Holocene as a consequence of ice retreat which occurred at the end of the last glaciation (Bertolini et al. 2004). Consequently, most landslides reactivate at slow velocity while the frequency of first-time landslides is very low.

### Hazard assessment

In this work, the “hazard” component of risk is considered the spatial probability of a landslide occurrence and is thus approximated with an index derived from previous landslide susceptibility studies. Since landslide susceptibility mapping is a complex task and the focus of the work is the establishment of a more general procedure to quantitatively assess the risk at the national scale, the present study relies on an already published slow-moving landslide susceptibility map of Italy (Trigila et al. 2013). The map was obtained by applying the random forest treebagger (Breiman 2001; Brenning 2005; Catani et al. 2013), a machine learning algorithm widely consolidated in LSM studies (Catani et al. 2013; Kavzoglu et al. 2019; Xiao et al. 2020), to a series of environmental parameters (lithology, land cover, elevation, slope gradient, curvature, profile curvature, planar curvature, aspect, flow accumulation, topographic wetness index and stream power index). The algorithm was trained with the official national landslide inventory of Italy (IFFI), which is recognized as one of the most complete and homogeneous existing national databases (Trigila et al. 2010; Herrera et al. 2018). The peculiarity of the map is that the algorithm was not applied with a classical classification mode based on two classes (namely presence/absence of landslides), which is the most recurrent approach in the international literature. The approach of Trigila et al. (2013) was based on a regression model, trying to predict continuous values from 0 to 1, expressing the percentage of area interested by landslides. Its accuracy was evaluated by means of the AUC (area under the receiver operator characteristic curve), which was equal to 0.76, showing a relatively good result for susceptibility maps based on regression models.

The original susceptibility map, based on 50 × 50 m pixels, was imported in a GIS system, clipped to the test site, and overlaid on an updated version of the IFFI inventory. The hazard index was defined as equal to one in the pixels occupied by slow-moving landslides contained in the inventory and equal to the original susceptibility value elsewhere. Finally, an upscaling at the resolution selected for the present work was necessary: the values of the hazard index raster (with 50 m cell size) were averaged over each 1 km^2^ cell of the reference grid used for risk analysis, resulting in a spatial hazard index theoretically ranging from 0 to 1.

### Landslide intensity

The selection of proper intensity parameters is an important issue. Intensity is not meant to express the magnitude (in terms of volume or area) of a landslide but its destructive capability, leading to a certain degree of damage. For slow-moving landslides (large slides, rockslides and earthflows), intensity is generally expressed in terms of total displacement (Saygili and Rathje 2009), differential ground deformation (Negulescu and Foerster 2010) and the displacement rate (Mansour et al. 2011; Frattini et al. 2013), closely related to the expected degree of damage to urban settlements and infrastructures in general. In areas affected by slow-moving landslides, the landslide extent can be considered a reasonable proxy for landslide intensity (Guzzetti et al. 2005). Consequently, in the present study, the landslide intensity (*I*) in the Arno River basin was estimated as a function of the landslide velocity scale (*v*) and the landslide area classes (*a*), which represent the most suitable parameters.

1 $$I=f\left(v,a\right)$$

The IFFI catalogue has been used to obtain information on the landslide distribution and extension within the Arno River basin, while information on landslide velocity has been retrieved thanks to Sentinel-1 SAR (synthetic aperture radar) data, which enable the detection of slow movements on the order of centimetres per year. MT-InSAR (multitemporal-interferometric SAR) products have already been exploited by many authors to define the velocity of potential slow-moving landslides (e.g. Raspini et al. 2018). Cigna et al. (2013) employed an MT-InSAR-based matrix approach to evaluate the state of activity and the intensity of extremely to very slow landslides. Bianchini et al. (2017) presented a GIS-based procedure aimed at evaluating specific risk at the municipality level using satellite interferometry as a landslide intensity zonation tool. Lu et al. (2014), leveraging geostatistical tools, used the maximum velocity retrieved by the MT-InSAR dataset as an indicator to derive a landslide intensity map. Tofani et al. (2014) leveraged both satellite and ground-based radar interferometry for the zonation of the intensity of a large complex landslide in the Northern Apennine (Italy). Solari et al. (2020) proposed using satellite interferometric data as a direct estimation of landslide intensity and as a proxy for the presence of unstable debris covers that could be the source areas of future debris flows, whose runout is foreseen by means of a basin-scale model. Despite these examples, the direct use of MT-InSAR information for landslide intensity estimation is still scarce, constrained for technical reasons to slow-moving landslides and limited, with few exceptions, to single slopes or small basins.

The spatial distribution of landslides within the Arno River basin has been investigated by calculating, for each cell of the grid, the landslide index, the most common tool used to assess the landslide distribution at a small scale. The index is calculated as the ratio between the landslide area within the cell and the extension of the cell itself (i.e. 1 km^2^). The values of the landslide index were classified into four classes (A_0_, A_1_, A_2_, A_3_) using the natural breaks (Jenks 1967) algorithm, which is a widely used method that maximizes variance between classes while reducing variance within each class. A0 is defined by a landslide index equal to 0; A1 for 0 < landslide index ≤ 14.30; A2 14.30 < landslide index ≤ 38.43 and A3 for landslide index > 38.43.

**Fig. 3:**
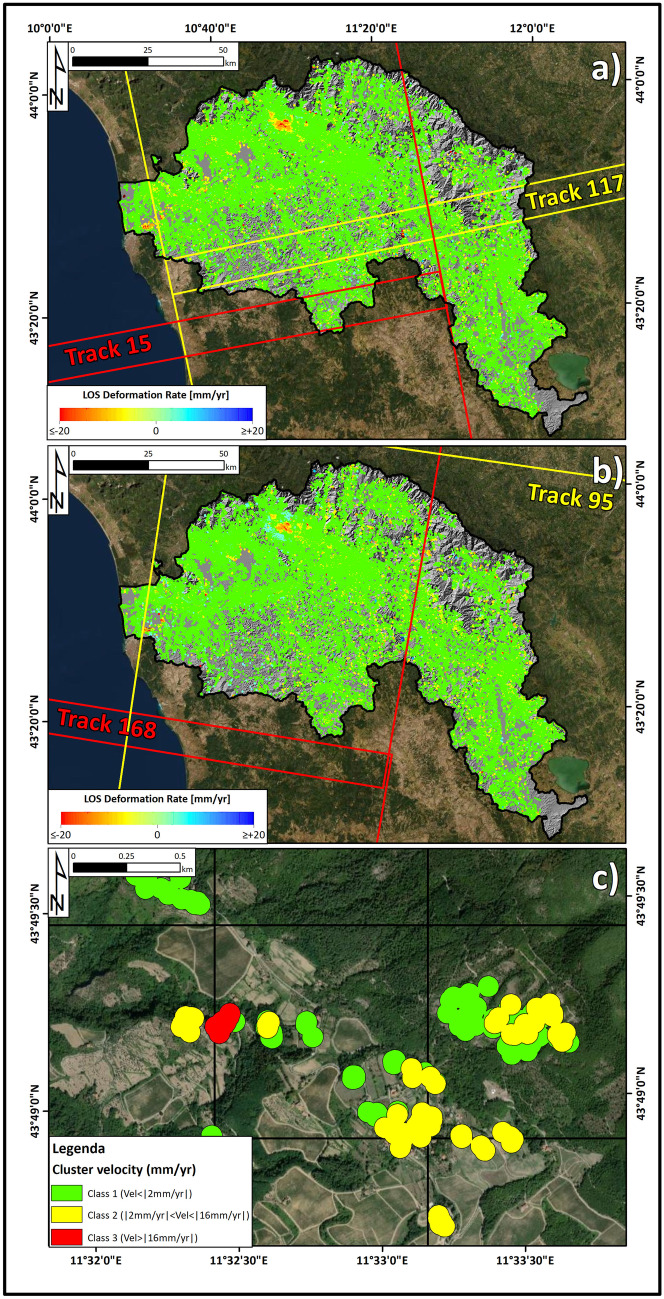
(**a**) Measurement points of ground deformation acquired in ascending geometry. (**b**) Measurement points of ground deformation acquired in descending geometry. (**c**) Results of the clusterization process

The spatial distribution of ground deformation induced by landslides has been retrieved for the Arno River basin by adopting a clustering-based method that allows for the extraction of moving areas from datasets containing millions of measurement points (MP) (Fig. 3c). The method, already followed by other authors (e.g. Barra et al. 2017), can be divided into two main steps: (*i*) filtering of the raw deformation map: MP was filtered with different velocity threshold values to separate areas with different deformation rates. A spatial criterion based on the variability of a point with respect to its neighbours was adopted to discard sparse measurements (isolated points) and points with strong discrepancy with respect to its neighbours (outliers); (*ii*) cluster extraction: the resulting filtered maps were further analysed by creating a buffer zone of 100 m around each MP. Only cluster sizes of at least 3 MS were considered representative. The buffer and cluster sizes have already been considered reliable by other authors (Montalti et al. 2019) on the basis of the extent of landslides in the Tuscany region. Each cluster is characterized by the mean velocity, standard deviation and coherence of the time series of the PS/DS points comprising the clusters.

The final output is a geodatabase of polygons containing information (rate, extent and TS coherence) on ground motion patterns at the basin scale. Two different velocity threshold values were set to define three different classes: Vel_1_ (velocity < 2 mm/year), Vel_2_ (2 mm/year < velocity < 16 mm/year) and Vel_3_ (velocity > 16 mm/year), with velocity values expressed in absolute value. The reason 16 mm/year was selected as the threshold to separate Vel_3_ and Vel_2_ is that it corresponds to the lower velocity boundary of an active slow-moving landslide (1.6 m/year), according to the classification proposed by Cruden and Varnes (1996). Similarly, 2 mm/year was chosen as the boundary between Vel_2_ and Vel_1_ because it corresponds to the stability interval of the interferometric techniques, set on the basis of the statistical velocity distribution and the standard deviation of the Sentinel-1 dataset. A fourth class has been added (Vel_nd_), representing those areas lacking measuring points and where information about the ground deformation rate is missing and which therefore represent a separate class. Cluster polygons intersect with the grid to associate a velocity value with each 1 km^2^ cell.

From both the IFFI database and the Sentinel-1 clusters, landslide and cluster polygons with an overlap lower than 1 ha with the grid cell were discarded and not considered in the analysis. The degree of landslide intensity for different combinations of landslide velocity and area can be represented in the form of a landslide intensity matrix. The landslide intensity matrix with different combinations of landslide area and velocity encompasses five classes (from I0 to I4, i.e*.* null, low, moderate, high and very high) (Table 2).

**Table 2:** Contingency matrix of the landslide intensity

		**Cluster velocity (mm/year)**			
		**V**_**1**_	**V**_**2**_	**V**_**3**_	**V**_**nd**_
**Landslide index**	**A**_**0**_	I0	I1	I2	I0
	**A**_**1**_	I1	I2	I3	I1
	**A**_**2**_	I2	I3	I4	I2
	**A**_**3**_	I3	I4	I4	I3

Intensity classes were used in the following phases for the choice of physical vulnerability values. In slow-moving landslides, people are not usually endangered, but damage to buildings and infrastructures might be high. Affected buildings may have structural or operational failures due to differential settlement or absolute displacement. In some cases, the accumulation of slow movement can lead to partial or total disruption (Del Soldato et al. 2019).

### Vulnerability

#### Buildings vulnerability

**Fig. 4:**
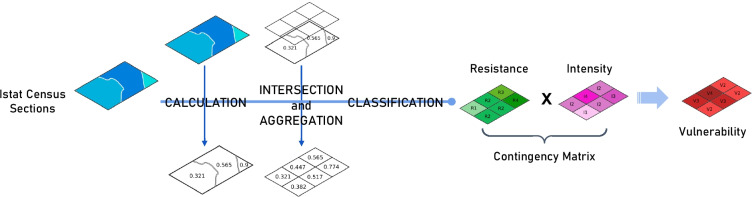
Flowchart of the building vulnerability assessment

Building vulnerability has been defined as a function of the structural resistance of buildings and the intensity of landslides. The vulnerability definition relies on a modified approach for the definition of structural resistance proposed by Li et al. (2010) and is based on the data of census sections produced by ISTAT in 2011. The procedure reported in Fig. 4 is constituted by three phases:

The first phase was the calculation of the structural resistance for each census section in the Arno River basin using a modified equation from the equation proposed by Li et al. (2010):

2 $${\mathrm{R}}_{\mathrm{str}}={\left({\varepsilon }_{\mathrm{sty}}\cdot {\varepsilon }_{\mathrm{smn}}\cdot {\varepsilon }_{\mathrm{sht}}\right)}^{1/3}$$

where ε_sty_, ε_smn_ and ε_sht_ are resistance factors of structure type, maintenance state and the number of floors, respectively. These parameters are derived from the census section data (Table 1). Census sections represent a homogeneous portion of the Italian territory, consisting of a single municipality, a portion of it or groups of municipalities characterized by similar environmental and socioeconomic characteristics. For each census section, useful information is provided, including building characteristics (e.g. typology, number, material, etc.) and population distribution (e.g. total number, number of families, etc.). For the computation of structural resistance, the resistance factors of Eq. 1 have been defined according to Li et al. (2010) and Uzielli et al. (2015) (Table 3).

**Table 3:** Resistance parameters and their values (from Li et al. 2010) that have been employed in the structural resistance assessment

**Resistance parameter**	**Resistance factor**	**Typology (from ISTAT census sections)**	**Value**
ε_sty_	Structure type	Productive and commercial	0.1
		Residential with light structure	0.2
		Residential with brick walls	0.8
		Residential reinforced concrete	1.5
ε_smn_	Maintenance state	Productive and commercial	0.1
		Residential in very poor condition	0.1
		Residential in a medium condition	0.6
		Residential in a good condition	1.2
		Residential in a very good condition	1.5
ε_sht_	Number of floors	Productive and commercial	0.1
		Single-storey residential	0.1
		Two-storey residential	0.4
		Three-storey residential	0.9
		Number of floors ≥ 4	1.5

The second phase of the procedure consists of the reaggregation of the structural resistance values computed for the census sections at the resolution of the analysis (cells of 1 km^2^) through a weighted procedure. Then, the cells were classified into six classes of structural resistance (Table 4).

**Table 4:** Contingency matrix of the building vulnerability

**Resistance**	**Intensity**				
	**I0**	**I1**	**I2**	**I3**	**I4**
**R4**: R_str_ > 1	0	0	0.25	0.25	0.50
**R3**: 0.7 < R_str_ ≤ 1	0	0.25	0.25	0.50	0.50
**R2**: 0.5 < R_str_ ≤ 0.7	0	0.25	0.50	0.50	0.75
**R1**: 0.3 < R_str_ ≤ 0.5	0	0.25	0.50	0.75	0.75
**R0**: 0 < R_str_ ≤ 0.3	0	0.25	0.75	0.75	1
**N.A**.: R_str_ = 0 or R_str_ = N.A	N.A. (0)	N.A. (0)	N.A. (0)	N.A. (0)	N.A. (0)

The third phase was characterized by the definition of the building vulnerability values for each cell; these values were determined through a contingency matrix that linked the landslide intensity classes with the structural resistance classes (Table 4). The contingency matrix defines five classes of vulnerability: V0 (null vulnerability), V1 (low vulnerability), V2 (medium vulnerability), V3 (high vulnerability) and V4 (very high vulnerability). To obtain quantitative values of vulnerability for the computation of the quantitative risk, the vulnerability classes were associated with numerical values ranging from 0 to 1 as follows: *V*0 = 0, *V*1 = 0.25, *V*2 = 0.5, *V*3 = 0.75 and *V*4 = 1. These values were defined with a calibration procedure largely based on empirical evidence collected during field surveys and detailed in the section “Calibration of the risk components”.

#### Land use vulnerability

Land use vulnerability assessment was based on the information given by the CORINE — Land Cover (CLC) database in 2012 (Table 1). This database gives a land cover map for the entire Italian territory and it can be obtained from the ISPRA website (Table 1). For this research, the CORINE land cover database was reclassified to have five classes of land use. A value of vulnerability, from 0 to 1, has been defined for each new land use class, according to the landslide intensity class of the reference cell. Then, for each cell of the grid, the weighted average value was calculated. The vulnerability values of land use are based on a previous study on landslide risk assessment in the Arno River basin (Catani et al. 2005; Lu et al. 2014) and by empirical evidence collected during field surveys, as explained in the forthcoming section, based on the calibration of the procedure.

### Exposure

In the landslide risk assessment studies, exposure (E) is commonly defined as the number or the value of the elements at risk (Schuster and Fleming 1986). Similar to vulnerability, in this study, vulnerability was assessed separately for buildings (E_b_) and land use (E_lu_).

#### Building exposure

To assess Eb, two national-scale open access datasets were selected: (*i*) OMI (*Osservatorio Mercato Immobliare* — real estate market observatory), managed and updated every 6 months by the National Revenue Agency; and (*ii*) Open Street Map (OSM), a collaborative and editable digital map of the world, where the spatial distribution of the buildings can be easily retrieved (Table 1). OMI data include the minimum and maximum trade values (expressed in €/m^2^) for the different typologies of the buildings, aggregated at the municipality level and further detailed according to the smaller subdivisions (subzones) of each municipality. The test site is comprised of 166 municipalities, counting 640 subzones. OMI includes observations repeated every semester, and data freely accessible online are comprised of 8 observations, spanning from the first semester of 2016 to the second of 2019.

The multitemporal data of all of the municipalities included in the study area were downloaded, imported into a spreadsheet and processed to define, for each subzone, the average market value of two distinct building typologies: residential buildings and others (including commercial and productive buildings). In the GIS system, the two average values were associated with the polygons representing the subzones. In the same GIS project, subzones were overlaid by the Open Street Map database (Haklay and Weber 2008) to associate to each mapped building with the total economic value according to its typology and its areal extension. The last step was a spatial reaggregation, where E_b_ was defined over the 1 km^2^ cells representing the basic spatial units of the work.

#### Land use exposure

To define E_lu_, two national-scale open access datasets were used: (*i*) VAM (*Valori Agricoli Medi* — average agrarian values), an open access database published online by the National Revenue Agency that provides the average estimated price (in €/ha) of different typologies of land (particular emphasis is placed on different cultures and different forest management), differentiated for homogeneous areas called “agrarian regions”; and (*ii*) CORINE Land Cover (CLC), an open access pan-European map of land use/land cover, which provides information on the biophysical characteristics of the Earth’s surface with high thematic accuracy (44 classes).

The classes of the CLC map and the different typologies encompassed by the VAM database have been aggregated into the five classes already used for the definition of vulnerability (Table 5). Then, in a GIS system, a unitary price was assigned to each polygon representing the agrarian regions on the basis of the extension of each CLC class. Finally, calculating the weighted mean, the average economic value of E_lu_ was defined for each cell of the grid.

**Table 5:** Land use classification based on the CORINE-Land Cover classes

**CORINE-Land Cover classes**	**Land use classes**
2.1–2.3–2.4	Agricultural areas
2.2	Permanent crops
3.1	Woods
3.2–3.3	Grasslands
4–5	Water

### Quantitative risk assessment

The quantitative risk map has been produced on a continuous scale in which numerical values indicate the distribution of risk expressed by the probability of expected losses for the elements at risk (Peng et al. 2015). QRA is performed separately for each type of element (specific risk), and the results are then integrated into a map of total risk by combining all maps of specific risk (total risk). In this work, two specific risk maps were produced: a building risk map and a land use risk map.

The specific risk of the buildings has been computed:

3 $${\mathrm{R}}_{\mathrm{b}}\left(\mathrm{I}\right)=\mathrm{H}\cdot {\mathrm{V}}_{\mathrm{b}}\left(\mathrm{I}\right)\cdot {\mathrm{E}}_{\mathrm{b}}$$

where R_b_ is the expected economic damages for buildings, H is the susceptibility, V_b_ is the vulnerability of the buildings, and E_b_ is the exposure of buildings.

Specific risk of lands use has been computed:

4 $${\mathrm{R}}_{\mathrm{lu}}=\mathrm{H}\cdot {\mathrm{V}}_{\mathrm{lu}}\left(\mathrm{I}\right)\cdot {\mathrm{E}}_{\mathrm{lu}}$$

where R_lu_ is the expected economic damages for land use, H is the susceptibility, V_lu_ is the vulnerability to land use, and E_lu_ is the exposure to land use.

The total risk is the sum of the specific risks of building and land use:

5 $${\mathrm{R}}_{\mathrm{tot}}={\mathrm{R}}_{\mathrm{b}}+{\mathrm{R}}_{\mathrm{lu}}$$

### Calibration of the risk components

The main drawback of the methodologies based on relational matrixes is the risk of being affected by a relevant degree of subjectivity and uncertainty. In the proposed procedure, 13 distinct parameters (e.g. H, I, V, …) are taken into account; some of them are classified into classes, and some are combined into 3 matrixes. Obviously, the final result will be very sensitive to the subdivision into classes and to the way such classes are arranged together to define the matrix outputs. Consequently, a calibration procedure was necessary to reduce the subjectivity and uncertainty. The matrixes shown in the methodology section are the final version of a long process of calibration (involving the class break values and the input–output combination) that can be summarized as follows:

First, all matrixes were defined as leveraging, whenever possible, the literature criteria (the methodology sections provide references to the related works). However, most of the steps of the procedures were novel, and reference works are lacking. In these circumstances, we resorted to the Delphi method (Rowe and Wright 1999; Cairns and Wright 2018) to draft a first tentative version of the matrix. Basically, the Delphi method consists of gathering a number of experts, interviewing them on the matter, and summarizing their expert judgement. Although widely used (Qing 2008; Hayati et al. 2013; Kaufmann 2016), this method is highly subjective; therefore, it was used only as a starting point for the real calibration procedure.

Later, all draft matrixes were calibrated in the field. An initial series of field surveys was carried out to check, at representative locations, the consistency of the matrixes with the field evidence. The sites were selected based on the occurrences of the matrixes according to two priorities: (a) some matrix combinations needed to be refined because the Delphi method resulted in divergent expert judgements; (b) in some spatial units, unreasonable final or intermediate results were obtained, highlighting the need to recalibrate some steps of the procedure. The site investigations were performed either in long campaigns during a single day over a relatively wide area (a 15 km^2^ rectangular block composed of 3 × 5 spatial units) or in more focused investigations on a smaller area (usually one or two spatial units). In both cases, the site was surveyed, checking all of the passages of the procedure, highlighting the discrepancies with the observed field evidence and hypothesizing corrections during the calibration process.

For the recalibration of the procedure, we also selected all sites inside the study area where the personnel involved in this work had recently worked in the framework of other site-specific research projects on landslide risk. Detailed reports were available, and the sites had already been surveyed in the recent past. This “delayed” recalibration provided additional data to corroborate the recalibration proposal coming from the former step of the calibration procedure. This procedure continued until all matrixes were supported by at least 2 convergent pieces of evidence collected in different locations and all contrasting hypotheses were proven wrong or less reliable.

To provide an independent validation of the matrix relations, another series of faster field surveys was carried out to check if the calibrated matrixes were also valid in different settings. Since this last check substantially confirmed the matrix calibration as described in the methodology section, we considered this version as the final version, and we adopted it to perform the quantitative risk assessment.

**Fig. 5:**
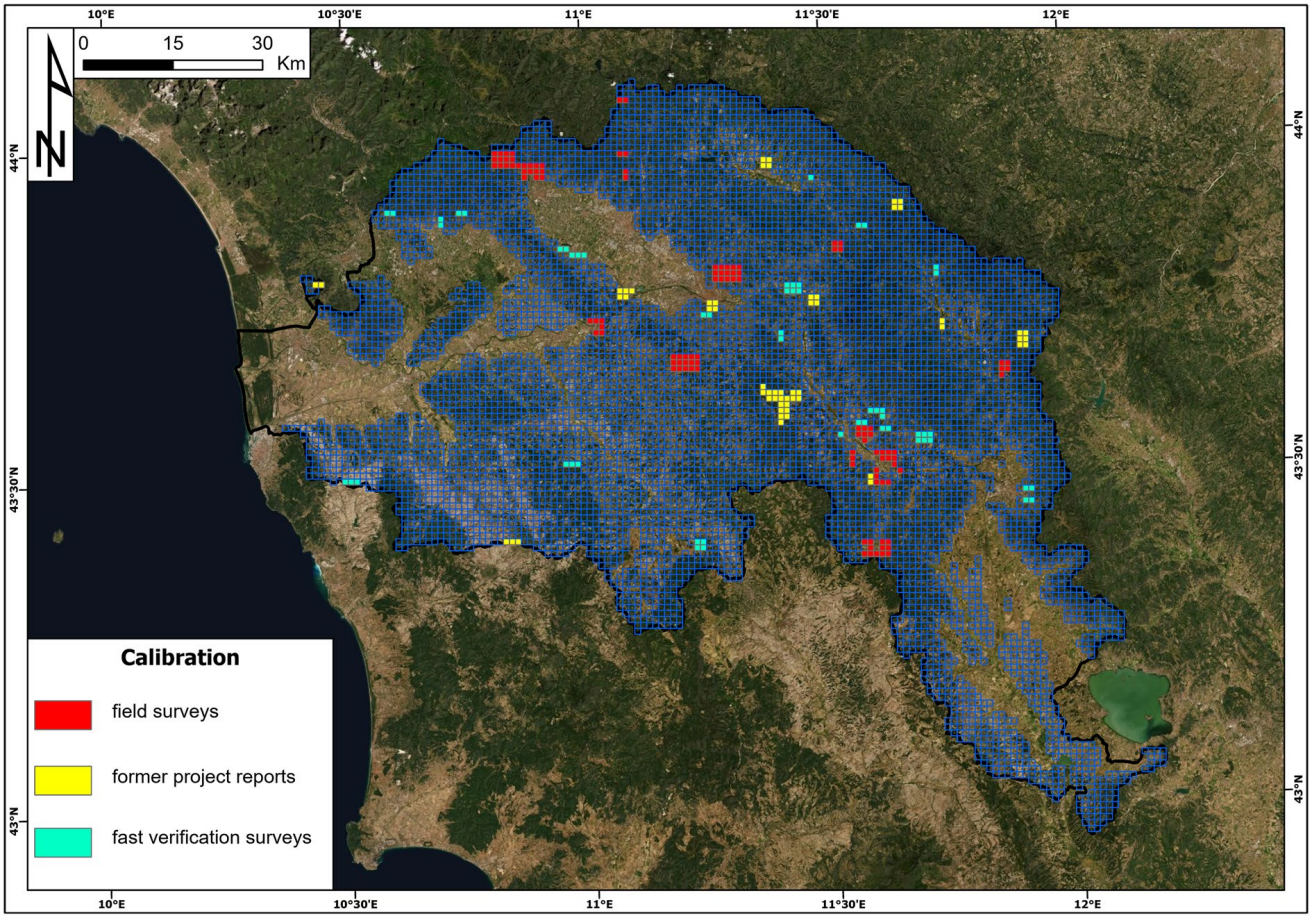
Location of the site investigation for the calibration process

**Fig. 6:**
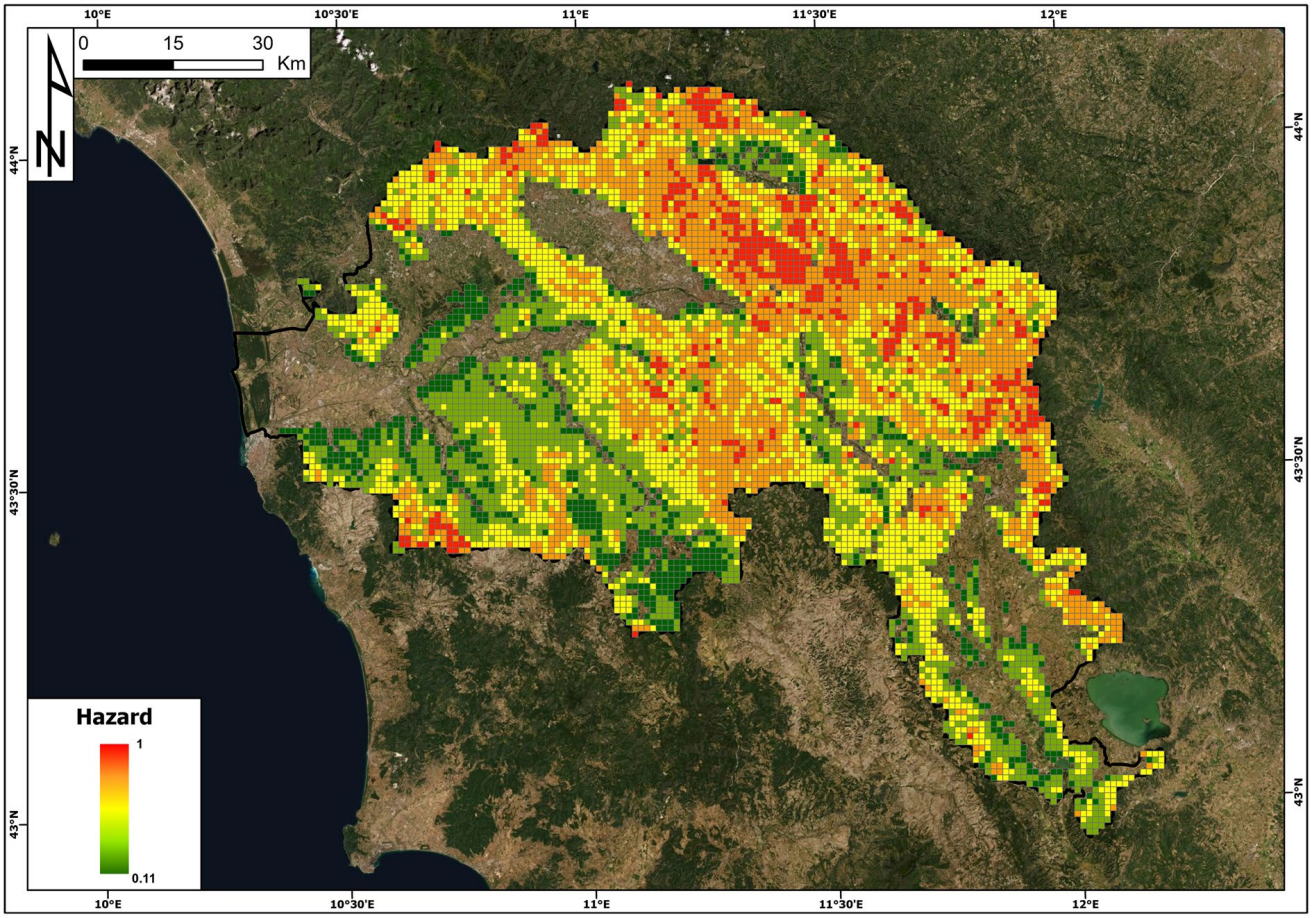
Landslide hazard map in the Arno River basin

On the whole, 129 spatial units were directly investigated on site, 59 were inspected indirectly, resorting to previous works (delayed surveys) and 70 were surveyed to validate the final version of the matrix calibration. From Fig. 6, it is clear that the pixels investigated are not evenly distributed. This is not necessarily a drawback, and it is due to several reasons: sites for direct inspections were not selected randomly or according to a regular survey grid but were selected based on the spatial location of the most problematic (e.g. uncertain or unreasonable results) matrix occurrences. Moreover, throughout most of the research project, movement across the test site was restricted due to the COVID-19 pandemic; consequently, surveys on spots away from the research team’s headquarters or residences were possible only during the last part of the fieldwork (fast validation surveys), where more homogeneous coverage of the test site was pursued (Fig. 5). Finally, the location of the “delayed survey” sites could not be selected since they corresponded to the test sites of former research projects.

## Results

### Hazard

The spatial distribution of hazards, defined as the spatial probability of landslide occurrence (cf. Methodology section), is reported in Fig. 6. Hazard values theoretically range from 0 to 1 but actually span from 0.11 to 1, as the areas where landslides are not expected as a geomorphological process (e.g. wide alluvial plains and flat areas) were filtered off to reduce the computation time. Landslide hazards are lower in the gentle hills occupying the south and west sectors of the area and in the areas surrounding the intermontane basins. From a comparison with Fig. 1, it is clear that the highest hazard values can be found in the main reliefs and are also influenced by the presence of active and quiescent landslides mapped in the IFFI inventory.

### Vulnerability

**Fig. 7:**
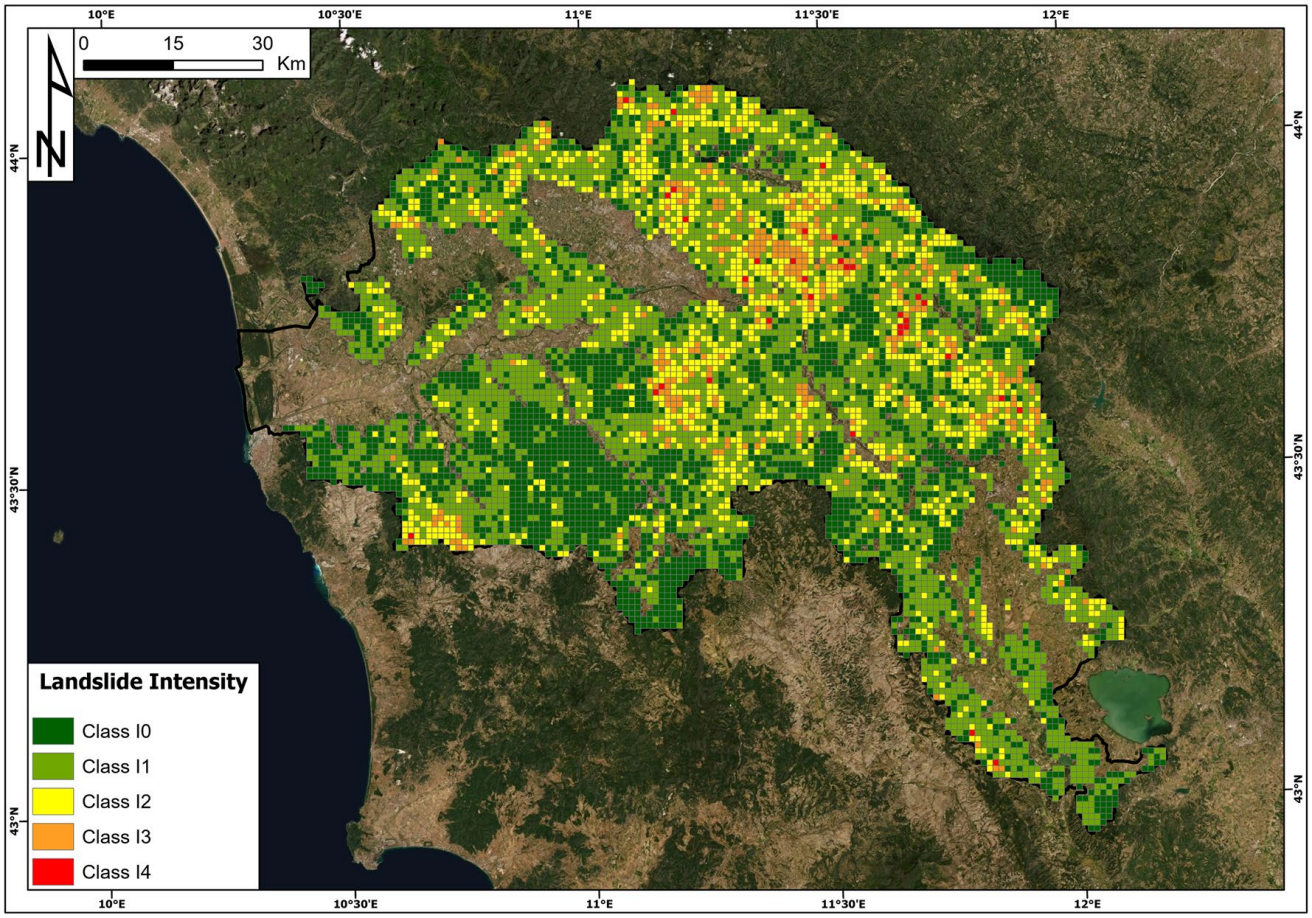
The landslide intensity map in the Arno River basin

The results of the intensity assessment are shown in Fig. 7 based on the classification reported in Table 2. The distribution of landslide intensity (Fig. 7) at the basin scale shows that the highest classes (I4 and I3) are more widespread along the Apennine Chain and in the Chianti area. The majority of 1 km^2^ pixels have an intensity class I1 (44% of pixels), followed by I0 (31%), I2 (19%), I3 (5%) and I4 (1%). Only 1% of the total number of pixels, corresponding to a total area of 34 km^2^, had the highest value of intensity (I4).

**Fig. 8:**
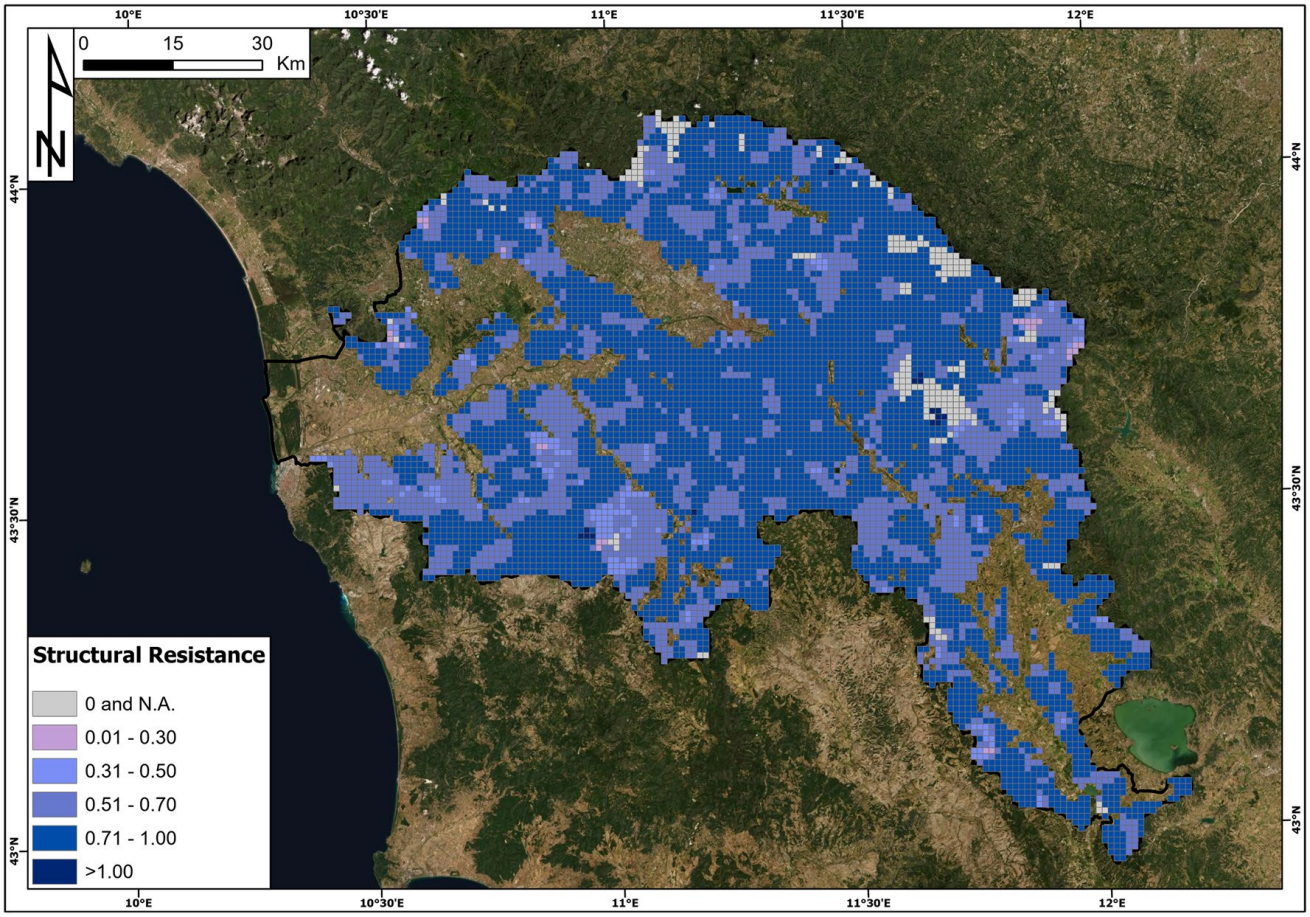
Structural resistance map in the Arno River basin

**Fig. 9:**
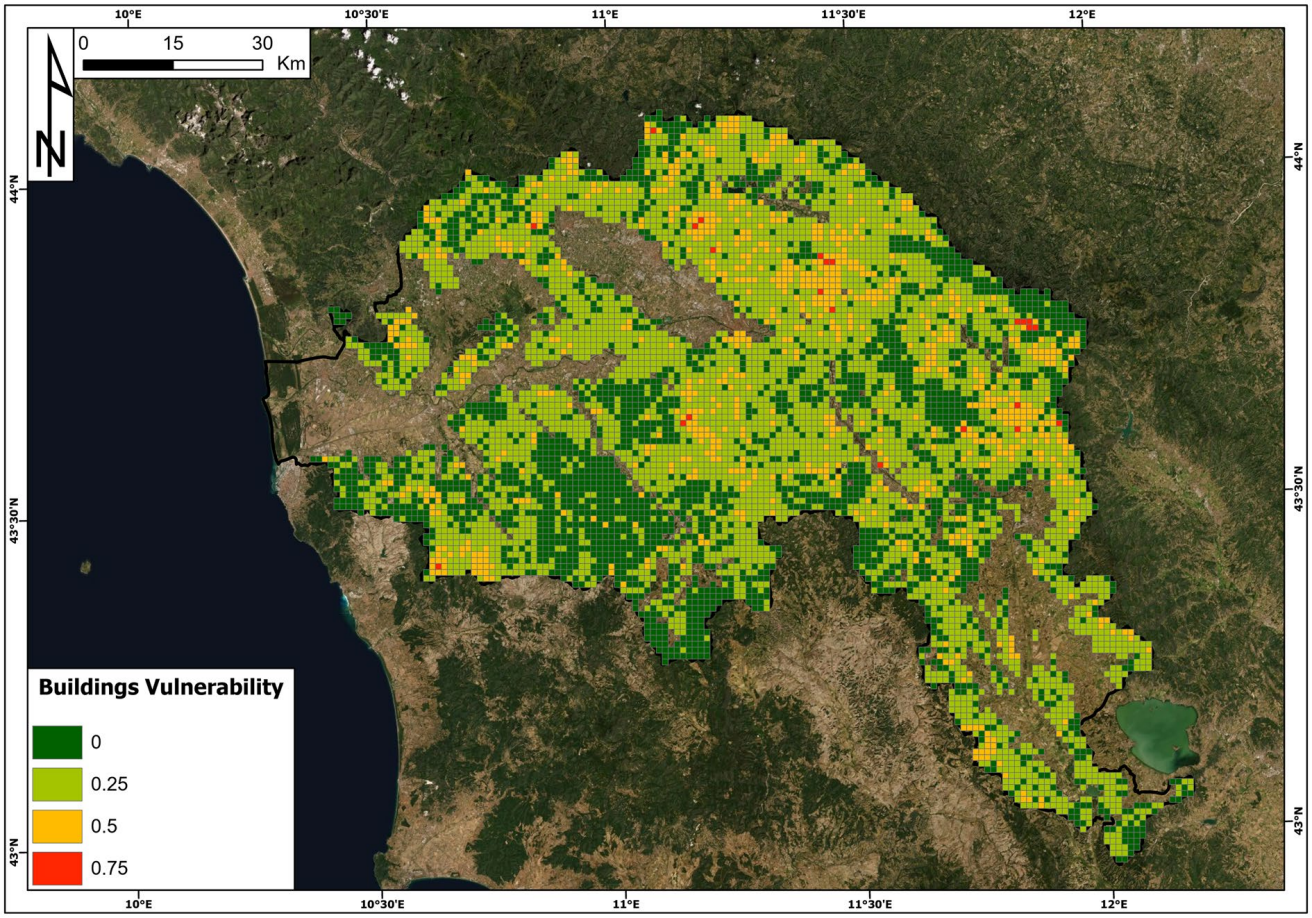
Building vulnerability map in the Arno River basin

Building vulnerability has been defined as a function of the structural resistance of buildings and the intensity of landslides based on the contingency matrix reported in Table 4. The results of building resistance, based on Eq. 1, are reported in Fig. 8, while the results of building vulnerability are reported in Fig. 9. Structural resistance is computed for each census cell and then aggregated at a 1 km^2^ resolution. The values of structural resistance vary from 0 (null resistance) to 1.3 (maximum resistance in the study area). The resistance values were classified into five classes: R0 (low resistance), R1 (low to medium resistance), R2 (medium resistance), R3 (medium to high resistance) and R4 (high resistance). Based on the resistance class percentages (R0: 0.38%, R1: 2.70%, R2: 29.06%, R3: 64.41% and R4: 0.25%), it is possible to conclude that the building resistance to slow-moving landslides in the study area is mainly medium to high. Due to unavailable information for some of the census sections, 3.20% of the cells had no building resistance information.

The building vulnerability (Fig. 9) is defined through a contingency matrix (Table 4) combining the landslide intensity with the structural resistance. In general, it is worth noting that the building vulnerability to slow-moving landslides within the Arno River basin is low, with a prevailing low vulnerability value (0.25). In detail, 33% of the cells have a vulnerability value of 0 (class V0), 54.7% have a vulnerability value of 0.25 (V1), 12% have a vulnerability value of 0.50 (V2), 0.3% have a vulnerability value of 0.75 (V3) and 0% have a vulnerability value of 1 (V4).

**Fig. 10:**
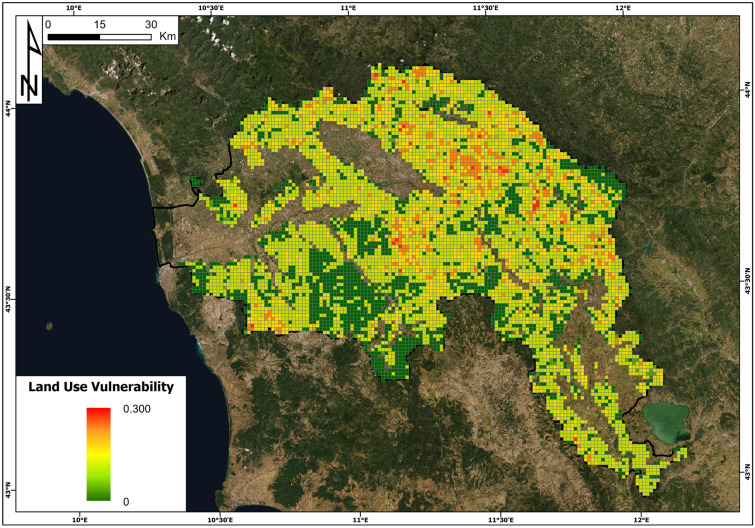
The land use vulnerability map in the Arno River basin

The vulnerability for different land use classes, as defined in Table 5, is reported in Fig. 10. The maximum value of vulnerability according to the contingency matrix in Table 6 is 0.3 and it occurs for the 1 km^2^ cell with the highest intensity (I4) and the presence of permanent crops and woods. The minimum value of land use vulnerability is equal to (0) and was verified with intensities I0, I1 and I2 for greenlands and water and with intensity I0 for all of the classes of land use. In general, based on previous studies, we can state that the land use vulnerability for slow-moving landslides can be considered lower than the building vulnerability.

**Table 6:** Contingency matrix of the land use vulnerability

**Land use classes**	**V (I0)**	**V (I1)**	**V (I2)**	**V (I3)**	**V (I4)**
Agricultural areas	0	0.05	0.1	0.15	0.2
Permanent crops	0	0.05	0.1	0.2	0.3
Woods	0	0.05	0.1	0.2	0.3
Grasslands	0	0	0	0.05	0.1
Water	0	0	0	0.05	0.1

### Exposure

**Fig. 11:**
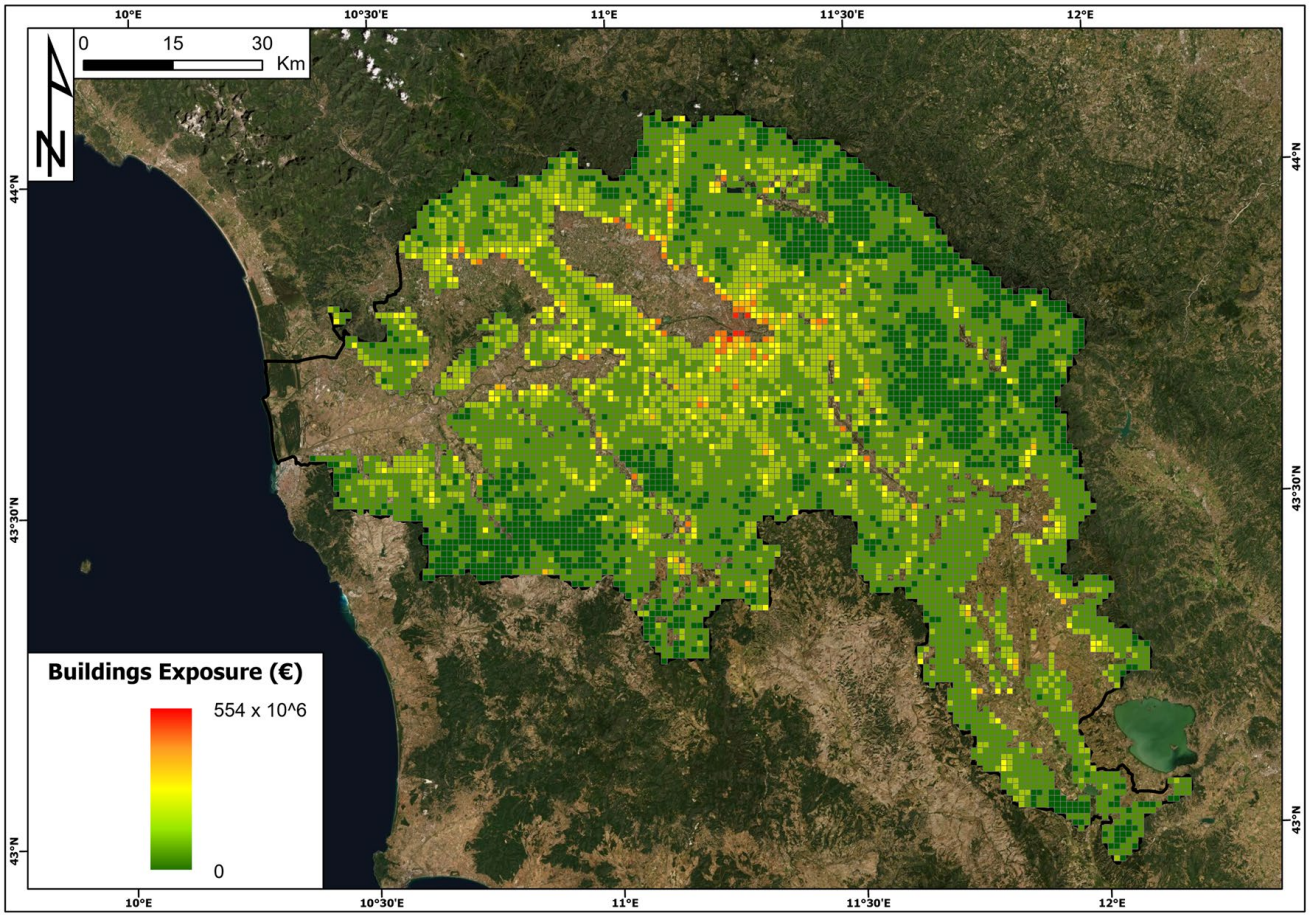
Building exposure (€) map in the Arno River basin

**Fig. 12:**
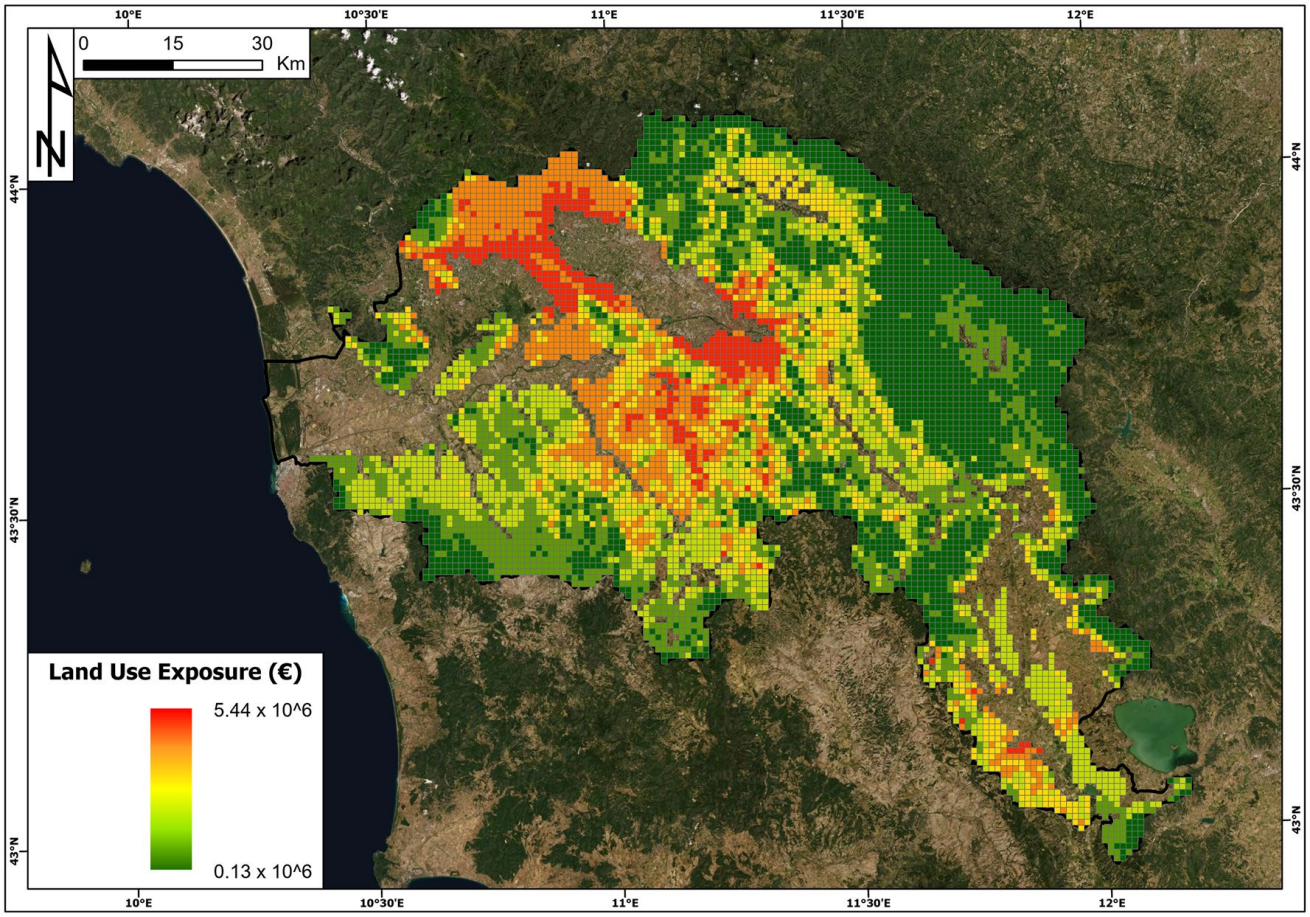
The land use exposure (€) map in the Arno River basin

The building exposure and land use exposure are reported in Figs. 11 and 12, respectively. Concerning building exposure, the maximum and minimum values per cell are 553 million euros and 0 euros, respectively. The average value per cell is 6 million euros, while the total building exposure is 56 billion euros. The cell with the highest value is located in the municipality of Florence. The cells with null values of building exposure were 19% of the total number of cells. Land use exposure has a maximum value of 5.4 million euros and is located in the province of Pistoia in the northern part of the basin. The minimum value is 0.134 million euros, the average value is 1.5 million euros, and the total sum of land use exposure is 10 billion euros.

### Risk

**Fig. 13:**
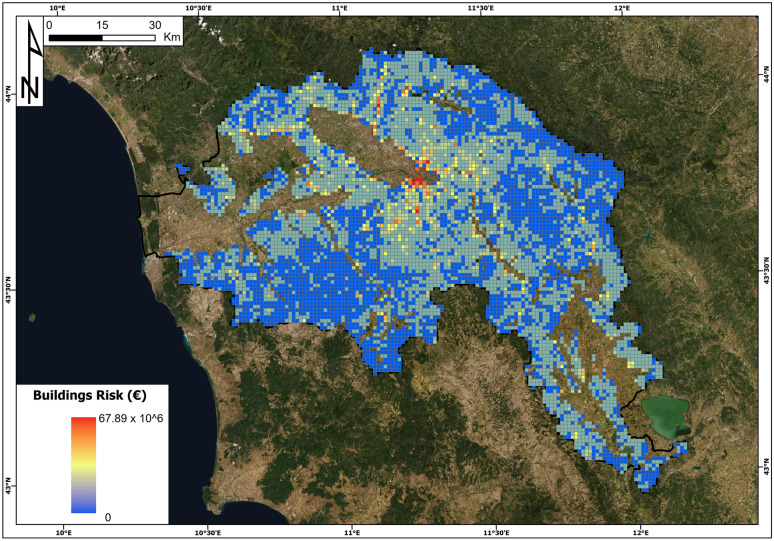
Landslide risk of buildings (euro) map in the Arno River basin

**Fig. 14:**
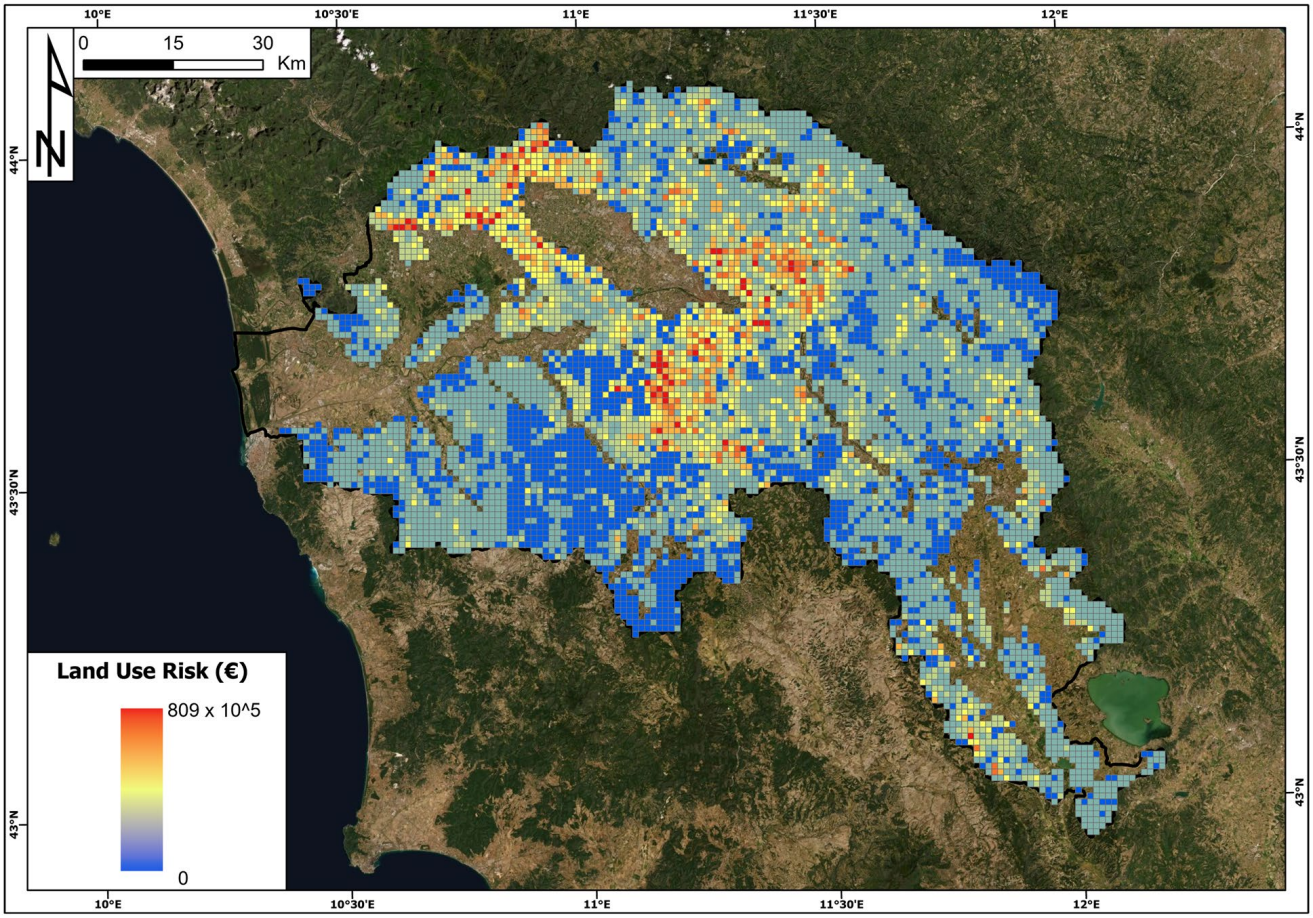
Landslide risk of land use (euro) in the Arno River basin

The buildings and land use risk maps are reported in Figs. 13 and 14.

The highest value of building risk is 67.89 million euros, and it is located in a cell of the municipality of Florence. The minimum value per cell is 0, while the average value is 0.896 million euros. The total value of the risk for buildings in the Arno River basin is 6.3 billion euros.

Concerning the land use risk, the cell with the highest value is in the municipality of Fiesole (near Florence), and its land use risk amounts to 0.809 million euros. The minimum value of this specific risk is 0, and the average is 0.050 million euros. The total land use risk is 0.35 billion euros.

**Fig. 15:**
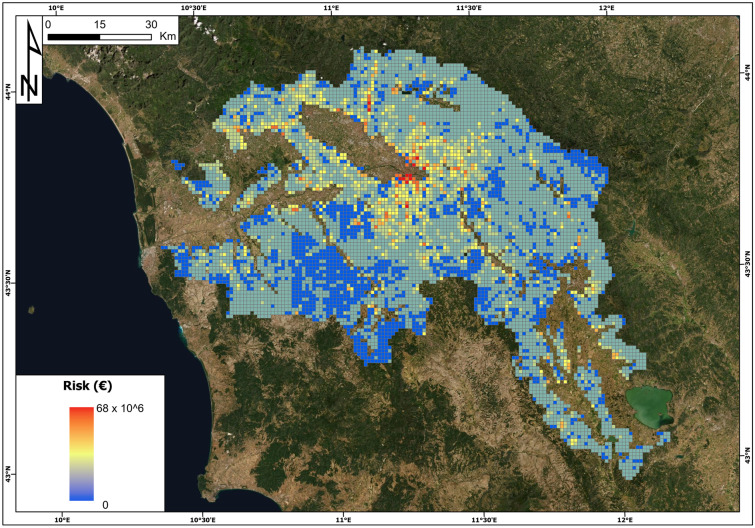
Total landslide risk (euro) map in the Arno River basin

The map of the total risk is reported in Fig. 15. The highest value is approximately 68 million euros, and the cell with this value is the same as the highest building risk. This cell reports the following parameters: hazard = 0.41, landslide intensity = I2, building vulnerability = 0.5, land use vulnerability = 0.09, building exposure = 405 million euros and land use exposure = 3.6 million euros. The minimum risk value in the study area is 0, the average is 0.946 million euros and the total sum is approximately 6.7 billion euros.

In detail, the cells with a risk greater than 1 million euros are 20% of the total number, and those with a risk greater than 10 million euros make up only 1.60%, mostly located within the municipality of Florence; likewise, the cells with a risk equal to 0 are 77% of the total.

## Discussion

### Discussion of the final results

In this work, a methodological framework for slow-moving landslide QRA analysis at the regional/national scale is presented. To test its applicability, we selected the Arno River basin (Central Italy), an area with an extension of 9100 km^2^ particularly affected by this landslide typology. The methodology leverages freely available open data at the Italian national scale to compute the different components of the risk equation with the final aim of obtaining a nationally reproducible and updatable map of landslide risk at the national level. The adopted scale of analysis is 1 km^2^.

For this reason, it is relatively easy to apply and implement common risk management practices. At the end of the procedure, for each cell defined over the whole territory of the Arno River basin, many kinds of information are available to support the activities of risk managers and local administrators, such as the hazard, intensity, vulnerability and the risk for buildings and land use (expressed in terms of economic losses). This framework represents a definite development in the field of risk management because it introduces an integrated and flexible approach for use by land planners and policy makers (Catani et al. 2005). Moreover, the proposed approach is scalable and could be upgraded to the national scale: this characteristic was the main idea behind the research approach and conditioned the development of all of the steps of the procedure.

An innovative part of the analysis is represented by the building vulnerability assessment. Generally, at small-scale analysis, such as regional or national analysis, building vulnerability is defined as 1 (highest loss) (Glade 2003; Uzielli et al. 2008), while in this paper, we have provided a semiquantitative process based on the approach proposed by Li et al. (2010) to assess building vulnerability at a small scale based on the information provided by ISTAT at the national level.

The obtained results show high values of landslide risk in the study area, with a total risk of 6.7 billion euros. Inspecting the first ten cells with the highest risk values, we discover that seven of them are located in the hilly portion of the Florence municipality where the exposure of the elements, with special reference to buildings, is very high. Building exposure has a relevant weight in the definition of the total risk: on average, building exposure is five times higher than land use exposure, and this difference is further amplified by vulnerability, which is typically higher for buildings than for land use: the specific risk of buildings is ten times higher than that of land use.

**Fig. 16:**
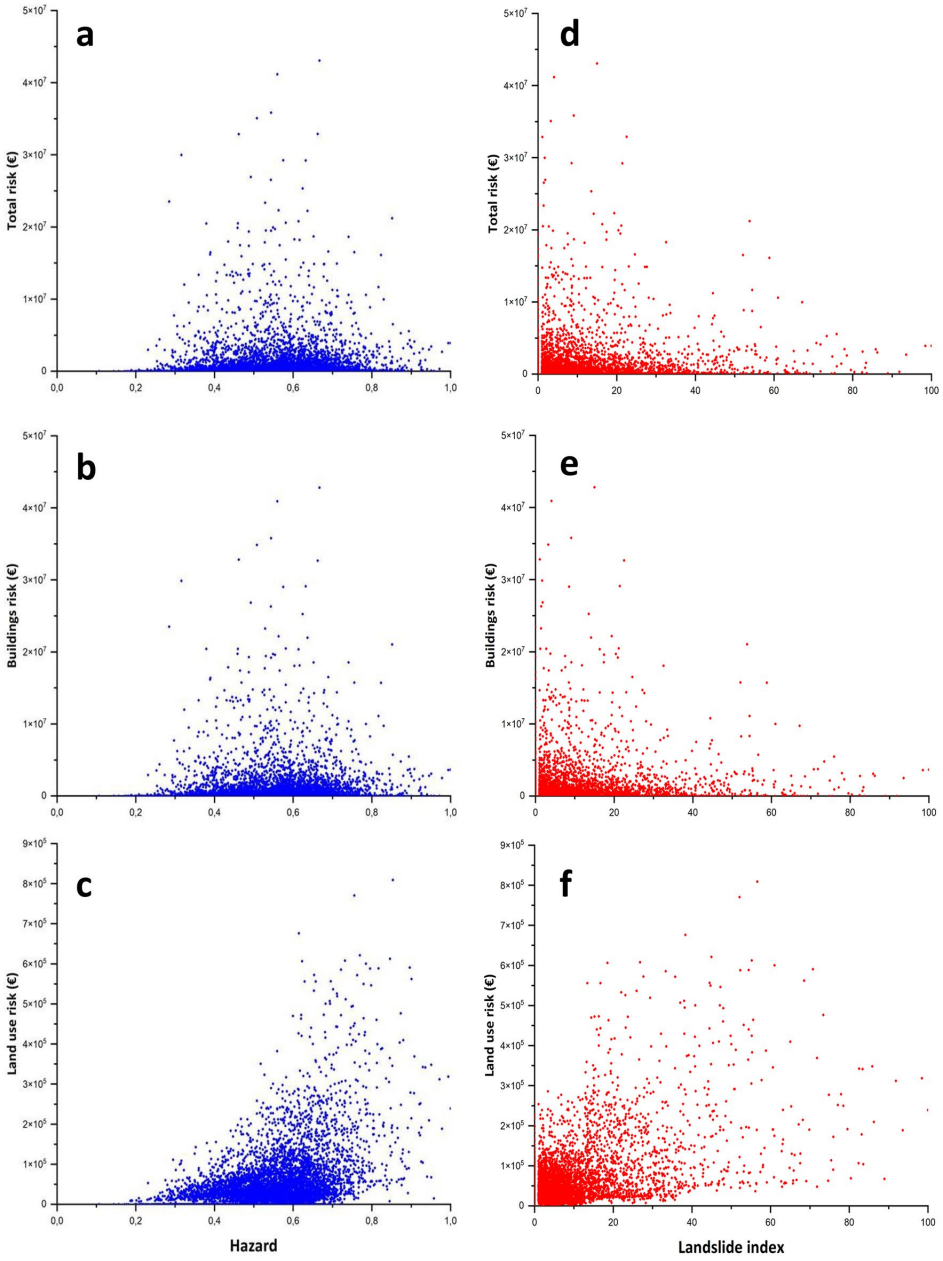
QRA values versus hazard and landslide index values. Total risk (panels **a** and **d)**; building risk (panels **b** and **e**); land use risk (panels **c** and **f**)

The finding that the building exposure has a fundamental weight in the risk values can also be observed in Fig. 16: in panels a, b and c, the hazard and risk values of the whole cells of the Arno River basin are compared, while panels d, e and f compare the values of the landslide index and risk. Concerning panels a, b and c, it is possible to observe that there is no relationship between the increase in hazard and total risk values and building values (Fig. 16a and b). There is instead a slight relationship between the land use risk and hazard, meaning that with the increase of the hazard, there is the increase of risk values related to land use. If we look at the comparison between the landslide index (percentage of landslide areas within each cell) and total and building risk, the situation is similar. The highest values of the landslide index do not correspond to the highest values of total risk and building risk (Fig. 16d and e). Nevertheless, it is possible to observe an increase in land use risk values with the increase in the landslide index. Moreover, the figure proves once more that the total risk value is dominated by the building risk (the same point patterns in panels a, b, d and e of Fig. 16).

Further insight into the patterns of the points in Fig. 16 reveals additional useful information on the results of the risk assessment. Panels b and e of Fig. 16 clearly show that there is no systematic correspondence between hazard and risk. This is not surprising, as good urban planning demands buildings to avoid hazardous areas (or areas where landslides are mapped). This is especially true, as in recent decades, national and regional laws have posed heavy building restrictions in the most hazardous areas. However, it should be noted that in the studied area, landslide hazard areas cannot be completely avoided, which would require directing building activities towards only flat plains, but in most cases, such areas are restricted since they are severely exposed to flood hazards. Iadanza et al. (2021) showed this concept very well for the whole Italian territory, and the Arno River basin is no exception.

These results of landslide risk assessment for the Arno River basin and the abovementioned analysis can lead to some general considerations and can highlight future analyses to be carried out: (i) the landslide QRA values related to buildings are much higher than for land use; (ii) the highest values of building risks per cells are not directly related to landslide prone areas, meaning that the most hazardous areas have been avoided by building activity; on the contrary, if the hazard or landslide index increases, land use risk increases, meaning in general that most hazardous areas are located far from building structures and that the values of building exposure in urban areas contribute to the risk values.

### Main limitations and perspectives of future improvements

Although the present methodology represents a step forward towards the application of QRA in very large areas, several issues remain unresolved. In this section, the main drawbacks are identified and discussed, providing some general insights on how they could be addressed in future developments of the research: the need to explicitly account for a time dimension in the analysis, a more thorough parameterization of the exposure, additional calibration/validation procedures and the impact of using more accurate input data to get to more reliable results.

**Fig. 17:**
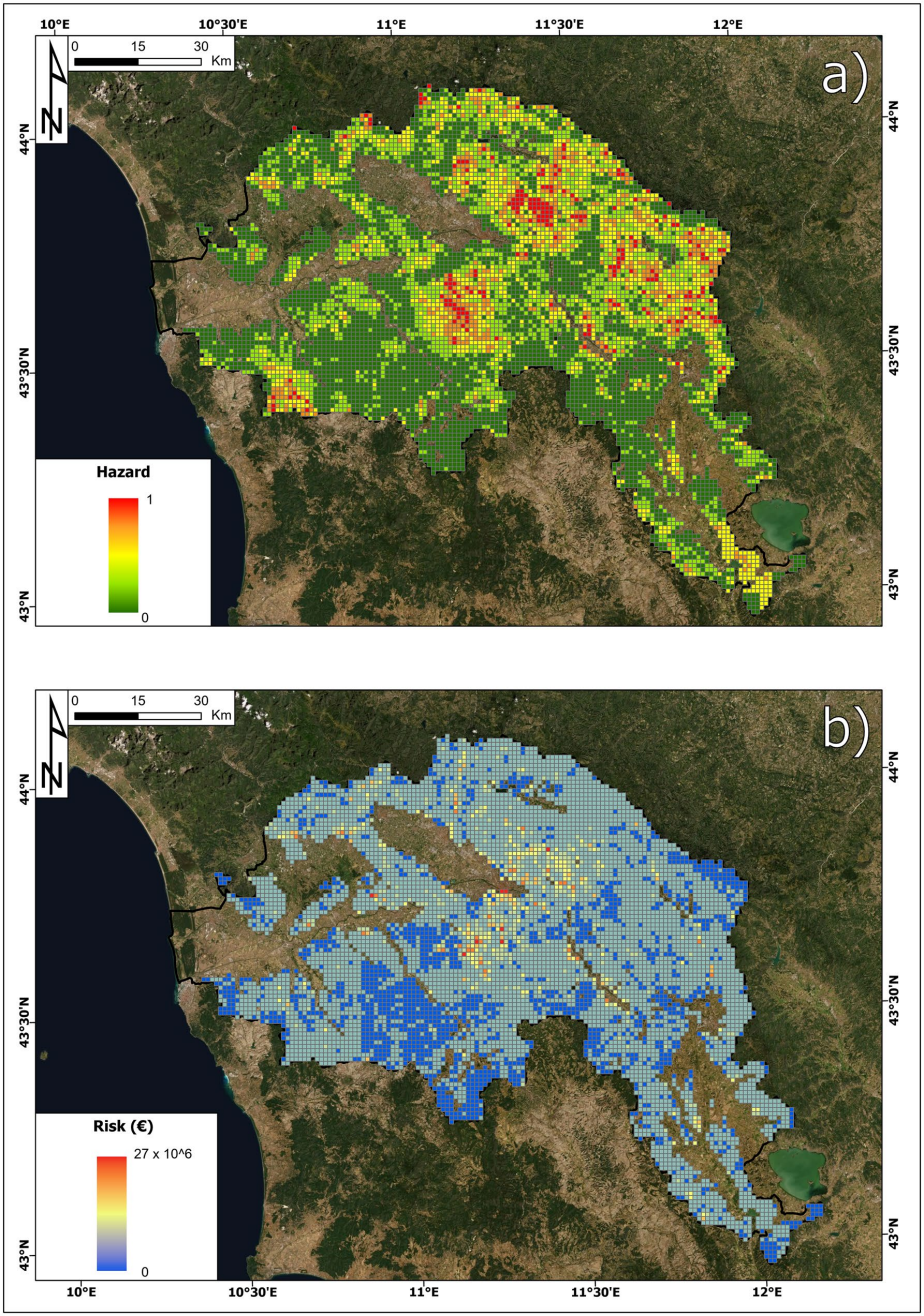
Hazard map based on the susceptibility map performed by Catani et al. (2013) (panel **a**); Total landslide risk map resulting from the above hazard map (panel **b**)

The result of every methodology is sensitive to the quality and accuracy of the input data, consequently it is important to check how the results are affected if different source data are used. In particular, we tested to what extent a landslide susceptibility map of higher accuracy can influence the results. In the previous part of the work, the susceptibility map developed by Trigila et al. (2013) was used because it is the only one available for the whole territory of Italy, despite the relatively low AUC values (0.76). We repeated the whole methodology using a susceptibility input data, another susceptibility map, developed by Catani et al. (2013) for the same test area of this work, which demonstrated a higher accuracy (0.85). The two maps are developed using the same methodology (Random Forest), similar model settings and the same original explanatory variables, but they have been calibrated and validated with different training datasets, extended over the whole Italy (301,000 km^2^) and over the Arno basin (9100 km^2^), respectively. While the map of Trigila et al. (2013) produces a result that is optimized according to the spatial distribution of landslides in the whole Italian territory (301,000 km^2^), the map of Catani et al. (2013) produces a result that is tailored on the characteristics of the Arno basin (9100 km^2^). The Arno basin has a complex geological and geomorphological structure, but it can be certainly considered relatively homogeneous, if compared to the whole Italian territory. For this reason, the ML result shows a higher accuracy in terms of AUC. By running again the proposed procedure with this more accurate susceptibility map, results change significantly (Fig. 17). The hazard is significantly lower (Fig. 17a): the mean value drops from 0.54 to 0.18 and the number of cells with very high hazard (i.e. > 0.75) drops from 3.8 to 1%. Of course, the large difference in the input susceptibility map influences all the subsequent steps of the procedure and produces a final risk map (Fig. 17b) that has a similar spatial pattern than the original one (Fig. 15) but consistently lower values: the mean risk in the cells of the map drops from 0.95 M € to 0.37 €, with low-risk values almost unchanged (the number of cells with zero risk value increases only by a 1%) and high-risk values drastically cut by the lower hazard values (e.g. the highest risk cell diminishes its value from 68 to 27 M €). Although the technical feasibility of the proposed framework is not in doubt, this test shows that for a reliable application at the national scale, more accurate input data (including a more trustworthy susceptibility map) should be used and, if not already available, prepared as a result of separate or associated research activities.

A structural limitation of the proposed procedure is that hazard is defined only in terms of spatial probability of occurrence, thus at the end of the proposed procedure, a static risk indicator is defined. Indeed, one of the main challenges of future research developments is to include in the procedure the evaluation of the temporal probability occurrence of landslides to get mid-term and long-term risk scenarios. At present, this task is hardly feasible at the national scale but with the ongoing developments in the fields of climate change characterization, statistical rainfall thresholds definition and setting up of updated and reliable landslide catalogues, we are confident that research progresses will be possible on this account.

Another shortcoming of the proposed procedure, which is common to most of the small-scale works on QRA, is that the definition of the expected impact of the natural hazards on the society depends mainly on the market value of buildings and land. This oversimplification may seem reasonable from the point of view of a landslide scholar, but it is very limited from the perspective of economics and econometry: the impacts of natural hazards may extend beyond direct damages or depreciation of buildings and may affect the probability of firms survival and their performances (with all connected indirect impacts) (Basker and Miranda 2018; Okubo and Strobl 2021). To obtain a more thorough assessment of landslide risk and possible impacts on the society, the next steps of the research will benefit from a closer collaboration between different fields of research to include econometric techniques in the definition of exposure.

Moreover, the next developments would include an improved calibration and a quantitative validation procedure considering information from different sources, including data on the financial expenses used for landslide risk mitigation in the Arno River to verify if the data retrieved in the analysis are reliable and sound.

## Conclusion

Landslides represent a worldwide natural hazard for human life and buildings, especially Italy, which is one of the countries with the highest exposure to hydrogeological risk. QRA plays a fundamental role in risk mitigation and management. The objective of this paper is to define and test a QRA methodological approach for slow-moving landslides in terms of expected damage to buildings and land use replicable at the national scale. The procedure was performed at a 1 km^2^ analysis scale and was tested in the Arno River basin, where most landslides are slow-moving. The risk assessment was based on the equation proposed by the Varnes and IAEG Commission on Landslides (1984), where risk = hazard × vulnerability × exposure.

The input data, aiming for national replicability of the methodology, are open and homogeneous for all Italian territories. Hazards were obtained by updating a previous susceptibility map (Trigila et al. 2013) with the IFFI database. Building vulnerability was determined through a semiquantitative process involving the structural resistance retrieved from the ISTAT data. Land use vulnerability was calculated thanks to an intersection between land use classes (CORINE-Land Cover database) and landslide intensity. Buildings and land use exposure were provided through average market values from OMI and VAM data, respectively. The parameters and the methods that we have used have been calibrated through field surveys or former project reports, which led us to the final versions of the procedures described in this paper.

The proposed procedure represents an advance in the application of QRA to very broad areas; nevertheless, the obtained results show a high value of total risk. For instance, the maximum risk calculated in a single spatial unit is 68 million euros, the total risk for the whole area is approximately 7 billion euros and the average value is 0.946 million euros. These high results are mainly due to the adopted scale of work, which has emphasized the great urban centres, so the buildings exposure is included in the risk assessment. Nevertheless, the proposed approach presents several novelties, such as the scale of the study, the possibility of replication at the national scale and the procedure for building vulnerability assessment. Concerning future developments, the definition of the “hazard” and “exposure” terms of the risk equation could be further improved, and a validation process must be carried out taking into account expenses related to mitigation measures of landslides.

## Data Availability

All input data used for this study are accessible online. Reference websites are indicated in Table 1.
